# Bacteriophages Improve the Effectiveness of Rhamnolipids in Combating the Biofilm of *Candida albicans*

**DOI:** 10.3390/molecules30081772

**Published:** 2025-04-15

**Authors:** Izabela Dusza, Dominika Jama, Grzegorz Skaradziński, Paulina Śliwka, Tomasz Janek, Aneta Skaradzińska

**Affiliations:** Department of Biotechnology and Food Microbiology, Faculty of Biotechnology and Food Science, Wrocław University of Environmental and Life Sciences, Chełmońskiego 37, 51-630 Wrocław, Poland

**Keywords:** *Candida albicans*, rhamnolipids, bacteriophages, antimicrobial agents, combined therapy, biofilm assay

## Abstract

Biofilms formed by *Candida albicans* pose therapeutic challenges due to their resistance to conventional antimicrobials, highlighting the need for more effective treatments. Rhamnolipids (RLs) are biosurfactants with diverse antimicrobial properties. Bacteriophages are viruses that target specific bacterial strains. Recent studies have shown that they may affect biofilm formation by fungi and yeasts. This study investigated the combined antimicrobial effects of RLs and bacteriophages against *C. albicans* biofilms, focusing on their anti-adhesive and inhibitory effects on biofilm development. RT-PCR assays were used to analyze gene modulation in *C. albicans* biofilm formation in response to RLs and bacteriophage treatments, while hyphae formation was examined using microscopy. The results showed that RLs-bacteriophage combinations significantly reduced biofilm formation compared to individual treatments. A combination of 200 mg/L RLs with bacteriophage BF9 led to a 94.8% reduction in biofilm formation. In a subsequent model, the same RL concentration with bacteriophage LO5/1f nearly eliminated biofilm formation (~96%). Gene expression analysis revealed downregulation of key biofilm-associated genes when *Candida* cells were treated with 200 mg/L RLs and four bacteriophages (BF17, LO5/1f, JG004, FD). These results show the potential of RL and bacteriophage combinations in combating *C. albicans* biofilms, presenting a promising therapeutic approach against resilient infections.

## 1. Introduction

*Candida* is a yeast pathogen that poses a range of threats to human health. Superficial candidiasis, such as oral thrush or vaginal yeast infections, can cause discomfort and affect the quality of life. Moreover, invasive candidiasis, particularly in immunocompromised individuals, can lead to bloodstream infections with high mortality rates. Additionally, the emergence of drug-resistant *Candida* strains presents challenges in treatment, potentially resulting in limited therapeutic options [1].

Biofilms produced by *Candida albicans* represent a particular risk to human health. These structures provide protection against host immune responses and antimicrobial agents, leading to persistent infections [2]. Moreover, *C. albicans* biofilms are associated with medical device-related infections, such as those linked to catheters and prosthetic implants, increasing the risk of complications and treatment failures. Additionally, the complex matrix of biofilms promotes the exchange of genetic material, contributing to the development of drug resistance, thereby further complicating treatment options.

However, given that *Candida* biofilms are notoriously resistant to antifungal agents, rendering treatment challenging and increasing the risk of recurrent infections, there is an urgent need for the development of new, effective, and safe strategies to combat infections of this origin [3]. Consequently, surfactants, renowned for their antimicrobial properties, are being investigated as potential solutions [4].

Surfactants are chemical compounds exhibiting surface-active properties [5]. Each surfactant possesses an amphipathic structure, characterized by a hydrophobic fragment (typically a hydrocarbon chain) and a hydrophilic fragment (consisting of anionic, cationic, non-ionic, or amphoteric groups). This structure enables these molecules to assemble into micelles, thereby diminishing interfacial and surface tension [6]. Surfactants can be synthesized through chemical processes, while biosurfactants are naturally produced by plants, animals, and microorganisms [7]. Biosurfactants are considered a more appealing option compared to synthetic surfactants due to their higher biodegradability, lower toxicity, superior emulsifying capacity, and foaming properties [5]. Their lower critical micelle concentrations (CMCs) contribute to enhanced efficiency in comparison to synthetic surfactants. Furthermore, these compounds are well-documented in the literature for their adhesive properties [8]. Biosurfactants find applications across diverse fields such as bioremediation, agriculture, and medicine, but also as pharmaceuticals, detergents, and cosmetics [9]. Rhamnolipids (RLs) are a type of glycolipids classified as low molecular weight biosurfactants alongside sophorolipids and trehalolipids. They consist of one or two rhamnose units acetylated with up to two hydroxy long-chain fatty acids. While predominantly produced by *Pseudomonas aeruginosa*, documented instances exist of other microorganisms capable of RLs production, such as *Burkholderia thailandensis* [10], or engineered strains of *E. coli* and *Pseudomonas putida* [11]. Typically, these compounds are present in a combination of mono- and di-RLs [12]. The specific composition of the RLs mixture can vary based on factors such as culture conditions, the carbon source utilized, and the bacterial strain involved [13].

On the list of alternatives to conventional antimicrobial strategies, the utilization of bacteriophages (phages, bacterial viruses) is frequently highlighted. Phages’ capability to eradicate bacteria has been harnessed almost since their discovery in the early 20th century. Phage therapy, once overshadowed by the advent of penicillin and the rapid advancement of antibiotic-based treatments, has experienced a resurgence in recent years as a response to the antibiotic resistance crisis. Phages possess several attributes that render them attractive for application. In contrast to biosurfactants, which influence the function of bacterial cell membranes, phages infiltrate cells and exploit the bacterial replicative machinery to proliferate, ultimately resulting in bacterial lysis [14]. Additionally, phages exhibit high specificity, targeting only specific types or even species of bacteria. Moreover, phages exhibit a unique characteristic termed “auto-dosing,” wherein they replicate at the infection site as long as the bacterial host persists. Finally, bacteriophages are deemed safe and non-toxic to humans and animals [15]. Although bacteriophages are believed to lack natural tropism for eukaryotic cells, interactions of such nature have been frequently reported [16,17]. Notably, these interactions extend to interference with yeast and fungal biofilm formation [18,19].

Current research is centered on developing combined systems to enhance pathogen elimination, with studies demonstrating, for instance, the synergistic effects of biosurfactants and antibiotics [20]. Therefore, this study aimed to investigate the combined effect of RLs and bacteriophages against *C. albicans* biofilms. The research assessed the formulations’ capacity to inhibit biofilm growth and prevent adhesion. To validate their efficacy, reverse transcription polymerase chain reaction (RT-PCR) was used to evaluate the expression of genes involved in *C. albicans* biofilm formation. Additionally, the ability of *Candida* cells treated with RLs, phages, and combinations of these agents to form hyphae was visualized with microscopy.

## 2. Results and Discussion

### 2.1. Critical Micelle Concentration (CMC)

**Figure 1:**
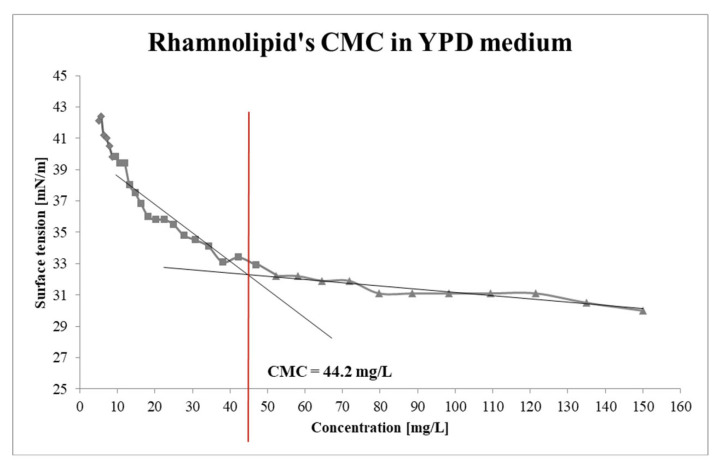
Effect of RLs concentration on surface tension: the critical micelle concentration (CMC) was determined by identifying the intersection point of the regression lines describing the two segments of the curve below and above the CMC.

The critical micelle concentration (CMC) is a pivotal parameter that describes surfactant compounds, signifying the minimum concentration of molecules requisite for achieving the lowest surface tension [5]. In elucidating the increase of anti-biofilm properties at concentrations above 50 mg/L, RLs surface tension was measured in a YPD medium by the du Noüy’s ring method (Section 3.2). Through measurements and curve plotting, the CMC for RLs was ascertained to be 44.2 mg/L (Figure 1). Noteworthy is the fact that biosurfactants remarkably attenuated the surface tension of the YPD medium from 42.1 to 30 mN/m, with concentrations of 5.15 mg/L and 150 mg/L of RLs, respectively, in contrast to the free of RLs YPD medium’s surface tension of 51.4 mN/m. Notably, even a minute concentration of RLs at 5.15 mg/L yielded a surface tension reduction of nearly 10 mN/m. This observation suggests that the mechanisms underpinning the antiadhesive activity of RLs against *C. albicans* may strongly hinge on micelle formation.

Literature posits that the CMC value of RLs is contingent upon the composition of homologs, particularly the ratio of di-RLs to mono-RLs. Li et al. determined the CMC for RL products derived from soybean oil and glycerol, revealing values of 65 mg/L and 50 mg/L, respectively. They juxtaposed these findings with a standard sample exhibiting a CMC of approximately 110 mg/L, containing almost three and two times fewer di-RLs compared to the test samples [21]. Hence, it can be inferred that the CMC value correlates with the di-RLs content in the mixture.

CMC values exhibit notable variability. For instance, Sharma et al. [22] reported a study wherein biosurfactants synthesized by *Pseudomonas aeruginosa* MTCC7815, utilizing waste cooking oil as the sole carbon source, displayed a composite of mono- and di-RLs with a low CMC of 8.8 ± 0.3 mg/L. Conversely, another study published in 2023 involved the production of RLs using *P. aeruginosa* FA1 in solid-state fermentation with peanut meal as the substrate. RLs obtained a CMC equal to 70 mg/L [23].

### 2.2. Influence of RLs on the Activity of Bacteriophages

To assess the potential inhibitory effects of RLs on bacteriophages, phages were subjected to incubation with varying concentrations of glycolipids (Table 1). A detailed statistical analysis of the results is presented in Table S1. Regardless of the concentration of RLs and the type of phage used, in all cases, the phage titer did not drop below the value of 1 × 10^8^ pfu/mL. The most notable reduction recorded was observed for bacteriophage LO5/1f. However, even in this instance, a considerable portion of viable viruses (2.75 × 10^8^ pfu/mL) was detected following incubation with RLs.

While numerous scientific reports highlight the inhibitory properties of biosurfactants against human viruses [24,25,26], to the best of our knowledge, there is a dearth of data regarding their impact on bacteriophages. Slightly more information can be gleaned concerning the influence of synthetic surfactants on phage activity. Interestingly, contrary to our observations, most reports suggest an adverse effect of surfactants on phages [27,28]. Nevertheless, Fister et al. [29] conducted an experiment wherein bacteriophage P100 was subjected to incubation with two surfactants, Lutensol (5%) and Sodium Dodecyl Sulfate (SDS) (5%), with the phage titer evaluated after 1 h, 6 h, and 24 h. The results indicated that while Lutensol did not alter the phage titer within a day, SDS caused a drop in phage titer of 1.2 log10 units. These observations suggest that the impact of surfactants on phage viability may depend on the type of molecule employed.

The impact of bacteriophages and RLs individually on the growth of *C. albicans* in liquid culture was also assessed (Figure S1). Only the RLs with the highest concentration of 200 mg/L significantly inhibited the growth of *C. albicans* compared to the control (*p* < 0.05) (Table S2).

### 2.3. Antiadhesive Properties of RLs, Bacteriophages and Their Combination

**Figure 2:**
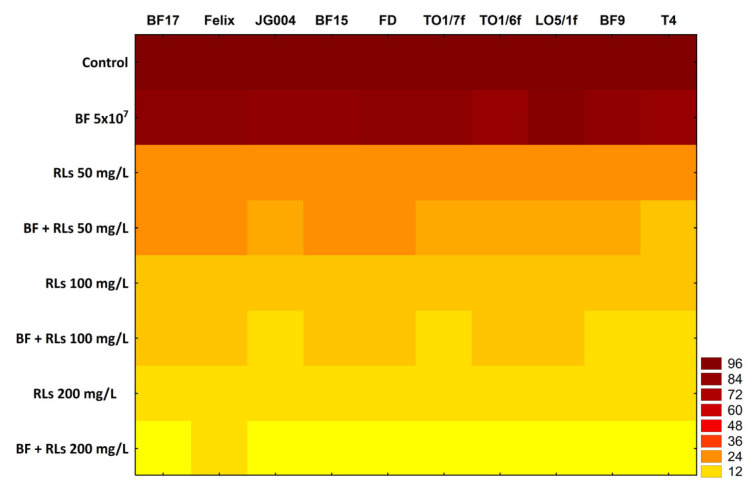
The percentage of biofilm formation by *C. albicans* on surfaces pretreated with RLs, bacteriophages, and their combinations. The results represent the averages of triplicate experiments. The figure includes a color scale indicating a percentage of biofilm formation.

The initial attachment of cells to a surface plays a pivotal role in the formation of a biofilm structure [30]. Consequently, many innovative approaches are directed towards thwarting biofilm formation by engineering anti-adhesive surfaces [31]. The findings of this study unveiled that RLs might impede biofilm formation by *C. albicans* in a concentration-dependent manner (Figure 2). These observations align with prior studies demonstrating the antiadhesive prowess of RLs in thwarting biofilm formation by *C. albicans* [32], as well as other microorganisms, including bacteria [33,34,35,36] and filamentous fungi [37].

Conversely, it is widely recognized that bacteriophages possess the capability to adhere to abiotic surfaces [37]. Furthermore, previous research has illustrated that this particular feature of phages could be involved in developing safeguarding surfaces against bacterial contamination. For instance, phages may be used in designing coating materials aimed at preventing biofilm formation on surfaces such as catheters, materials particularly prone to microbial infections [38]. The study’s outcomes indicated that despite the demonstrated inhibitory effect of phages in anti-adhesion treatments against *C. albicans* cells, the effect is notably weaker compared to that observed for RLs and a combination of phages and RLs (Figure 2). The tables with detailed results (Table S3) and statistical analysis (Tables S4 and S5) are provided in the Supplementary Material. The least inhibition of biofilm formation, a mere 4.9%, was noted for bacteriophage LO5/1f compared to the control lacking phages (*p* < 0.05). Conversely, the most robust inhibitory effect was observed for bacteriophages T4 and TO1/6f, which decreased biofilm formation by 17.7% and 19.6%, respectively, in comparison to the control (*p* < 0.05).

The primary objective of this study was to assess the antiadhesive properties of mixtures comprising RLs and bacteriophages in *C. albicans* biofilm formation. It is noteworthy that in all instances, irrespective of the RLs’ concentration and the type of phage used, combinations of both antimicrobials exhibited a stronger inhibitory effect against biofilm formation by *Candida* cells compared to when these factors were applied individually (*p* < 0.05). Particularly, the combination of 200 mg/L RLs with phage BF9 led to a 94.8% decrease compared to the control with an untreated surface. However, even at the lowest RLs concentration (50 mg/L), combination with phages resulted in approximately an 80% reduction in biofilm formation (ranging from 77.8% for phage BF15 to 85% for phage T4) (*p* < 0.05). Significantly, bacteriophage preparations undergoing the amplification process may contain bacterial residues stemming from host cell lysis. Therefore, to ascertain whether the observed synergistic effect was not influenced by bacterial fragments, bacterial lysates devoid of phages were prepared. Subsequent experiments were conducted analogously to those with preparations containing bacterial viruses (Section 3.6). No inhibitory effect on biofilm formation by *C. albicans* cells was observed with bacterial lysates, indicating that phages are indeed the agents enhancing the antiadhesive activity of RLs (Table S6).

### 2.4. Anti-Biofilm Properties of RLs, Bacteriophages and Their Mixtures

**Figure 3:**
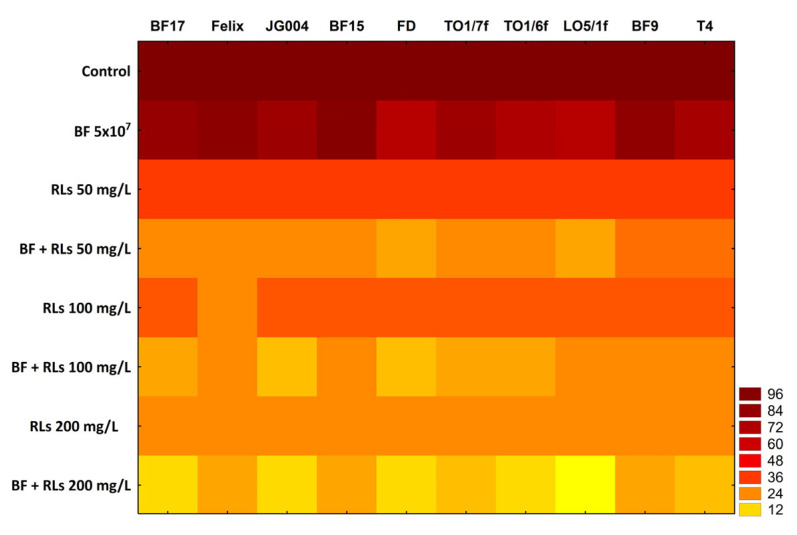
The percentage of biofilm formation by *C. albicans* in the presence of RLs, bacteriophages, and their combinations. The results represent the averages of triplicate experiments. The figure includes a color scale indicating a percentage of biofilm formation.

In an experimental model where RLs and bacteriophages were concurrently introduced into the wells alongside *C. albicans*, the outcomes mirrored those observed when antibacterials were utilized to pre-coat the surface to prevent biofilm formation. The tables with detailed results (Table S7) and statistical analyses (Tables S8 and S9) are provided in the Supplementary Material. Across all concentrations of RLs employed in individual experimental models, inhibition of *Candida* biofilm formation was evident (*p* < 0.05). However, for concentrations of 50 mg/L and 100 mg/L, the inhibitory effect was comparable, amounting to approximately 70% biofilm inhibition (Figure 3). Notably, at the highest RL concentration tested in the experiment, the inhibition was even more evident, reaching 77%. Bacteriophages exhibited a more pronounced inhibition of biofilm formation by *Candida* cells compared to the previous pre-adhesion model. Nonetheless, the effectiveness varied significantly among different phages. For instance, phage BF15 inhibited *Candida* biofilm formation by only 6% compared to the control (*p* > 0.05). Conversely, phages TO1/6f, FD, and LO5/1f demonstrated stronger inhibitory properties, reducing biofilm formation by 30.4%, 32.1%, and 35.5%, respectively, compared to the control (*p* < 0.05). Despite the limited scientific data on phage interactions with yeast cells, previous observations have indicated the inhibition of *C. albicans* biofilm formation by phages. Namely, Nazik et al. [18] demonstrated that phage Pf4, specific to *P. aeruginosa*, could inhibit *C. albicans* biofilm formation. This effect was correlated with the phage titer in the preparation, and notably, the phage was also effective in eliminating pre-formed biofilm.

Similar to the previous experimental model, synergy between RLs and phages was evident for each biosurfactant concentration and each phage tested. The most pronounced inhibitory effect was observed with the highest RLs concentration (200 mg/L) in combination with phage LO5/1f. This combination resulted in nearly complete inhibition of biofilm formation: approximately 96% compared to the control (*p* < 0.05). It is noteworthy that phages BF17, FD, and JG004 also significantly reduced *Candida* biofilm formation, achieving inhibition rates of 90.5%, 91.7%, and 91.8%, respectively.

### 2.5. Expression of Genes Responsible for Biofilm Formation by C. albicans in the Presence of RLs, Phages, and Their Combinations

Four experimental models demonstrating the most robust inhibition of biofilm formation were selected for the subsequent stage of the study, in which the synergistic effect of RLs and phages was analyzed at the molecular level. Four genes associated with biofilm formation were chosen, and their expression was assessed in the presence of RLs, phages, and their combinations. The *HWP1* gene encodes a cell surface protein exclusively expressed on hyphae, playing diverse roles, including cell wall assembly, intracellular signaling, hyphal development, and adhesion to epithelial cells. The products of the *ALS3*, *ECE1*, and *SAP4* genes, along with *HWP1*, contribute to hyphal growth [39,40].

**Figure 4:**
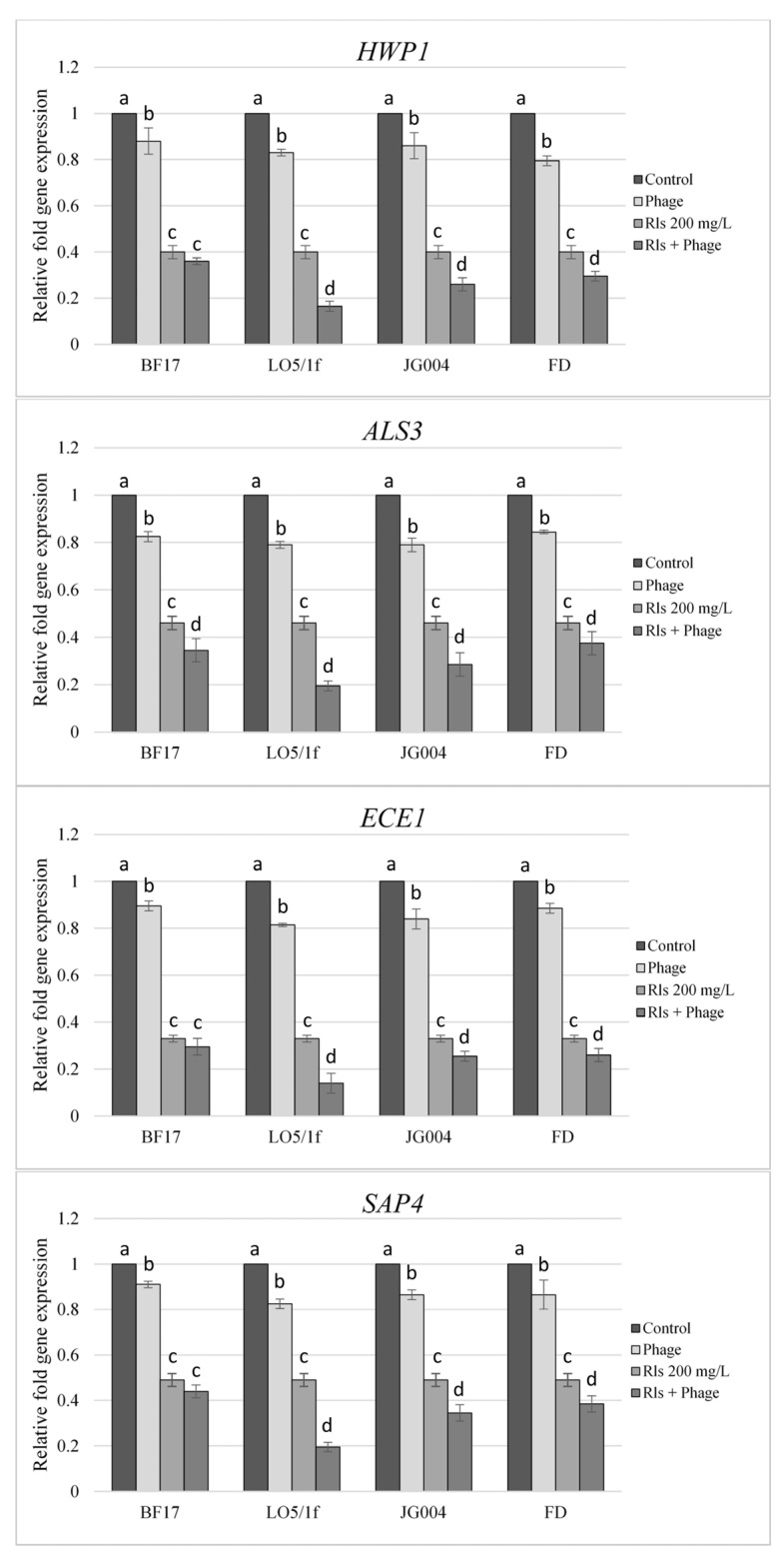
Expression of genes responsible for biofilm formation in *Candida* yeast treated with RLs, phages (BF17, LO5/1f, JG004, FD), and their combinations. Data are expressed as the means ± SD of the independent assays in triplicate. The same superscript letters designate homogenous groups comparing treatment samples within the same gene at a *p*-value of < 0.05.

The results showed that RLs may significantly reduce biofilm formation by downregulating the expression of genes related to this structure (*p* < 0.05). The results of the analysis are presented in the form of a graph (Figure 4), while the statistical analysis results are provided in the Supplementary Material (Table S10). This aligns with the findings of Haque et al. [41], who demonstrated that sophorolipids can inhibit the expression of hypha-specific genes *HWP1*, *ALS1*, *ALS3*, *ECE1*, and *SAP4* in *C. albicans*, possibly explaining the inhibitory effect of these compounds on biofilm formation. Although there are no analogous data on RLs, Saadati et al. [42] showed that RLs reduced the expression of quorum-sensing pathway genes *agrA*, *agrC*, *icaA*, and *icaD*, which are involved in the biofilm formation of methicillin-resistant *Staphylococcus aureus*. These data suggest that biosurfactants may influence biofilm formation by downregulating the genes responsible for this structure in various microorganisms.

On the other hand, no reports indicate that a similar mechanism operates in the case of bacteriophages, even for bacterial biofilms. The main mechanisms considered responsible for biofilm degradation by phages are as follows: (i) replication within cells and the spread of progeny bacteriophages through the biofilm, progressively removing cell layers; (ii) production of depolymerizing enzymes that degrade the EPS; (iii) induction of depolymerizing enzymes from within the host genome; and (iv) persistence in cells that, upon reactivation, commence a productive infection, thereby destroying the cells [43]. However, in this study, phages LO5/1f, JG004, and FD reduced the expression of all tested genes (*p* < 0.05), while phage BF17 downregulated the expression of *HWP1* and *ALS3* (*p* > 0.05). This suggests that influencing the expression of genes related to biofilm formation may be another potential mechanism by which phages interfere with biofilm, at least in some models.

Notably, the expression of each selected gene was significantly inhibited when *Candida* cells were treated with mixtures of RLs and bacteriophages compared to groups where biosurfactants and phages were used separately (Figure 4).

### 2.6. Microscopic Observation of Hyphae Formation by Candida Cells

**Figure 5:**
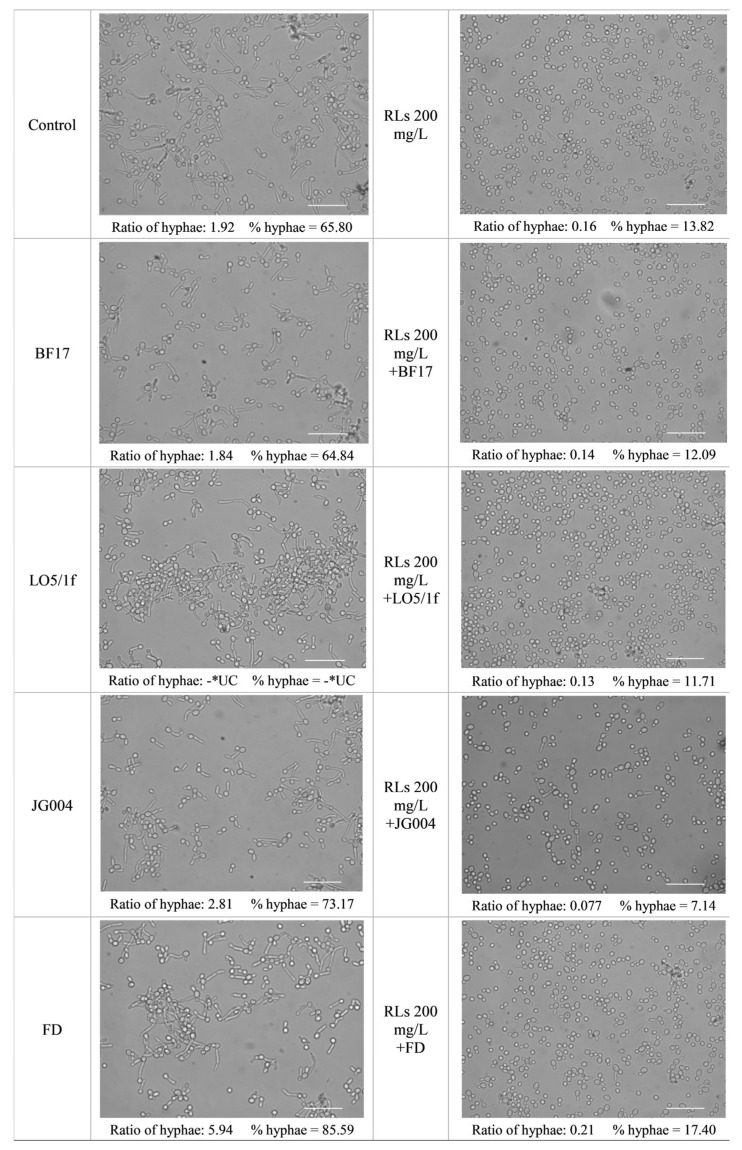
Effect of RLs, phages, and RLs/bacteriophages combinations on *C. albicans* mature hyphae. Scale bars = 50 µm. The ratio of hyphae (Ratio of hyphae) was determined as the number of hyphal cells divided by the number of yeast-form cells. The percentage of hyphal cells (% Hyphae) was calculated as the number of hyphal cells divided by the total number of cells (hyphal + yeast), multiplied by 100%. * UC—uncountable-cell counting is impossible due to extensive cell overlapping.

The synergistic effect of RLs and phages in inhibiting hyphae formation was subsequently validated through microscopic observation. Similarly, the same four models demonstrating the strongest inhibition of biofilm formation were selected for the experiments. Cells treated with mixtures of RLs and phages exhibited a significant reduction in hyphae formation compared to the control group comprising untreated cells, as well as in groups where RLs and phages were applied separately (Figure 5).

## 3. Materials and Methods

### 3.1. Strains, Media, and Compounds

The wild-type strain *Candida albicans* ATCC 10231 was used in this study. Frozen glycerol stock culture was regularly revived on YPD agar medium (yeast extract 10 g/L, peptone 20 g/L, glucose 20 g/L, and agar 20 g/L). The ATCC 10231 strain was cultured overnight in liquid YPD medium with shaking at 28 °C and then used for further experiments. RLs were purchased from AGAE Technologies LLC (Corvallis, OR, USA) as a mixture of different mono- and di-RLs homologs. The composition of the RLs sample is listed in Table S11.

### 3.2. Critical Micelle Concentration (CMC)

The measurements were carried out on a Krüss, K6 tensiometer (Krüss, Hamburg, Germany) using the du Noüy ring method. A 10 mL solution of RLs dissolved in YPD medium at a concentration of 150 mg/L was prepared. The surface tension of the YPD medium, RLs solutions, and their series of dilutions were measured. CMC was calculated by measuring the surface tension of RL solutions prepared in a YPD medium at different concentrations. Subsequently, a curve was plotted based on the obtained results, and two tangents were drawn. The point of intersection between these tangents determines the CMC.

### 3.3. Bacteriophages

Four bacteriophages, as well as their host bacteria (JG004/*P. aeruginosa*, Felix/*Salmonella*, T4/*E. coli*, FD/*E. coli*), were obtained from DSMZ-German Collection of Microorganisms and Cell Cultures (Leipzig, Germany). Six other bacteriophages (BF9/*E. coli*; BF15/*E. coli*; BF17/*E. coli*, TO1/6f/*Ent. faecalis*; TO1/7f/*Ent. faecalis*; LO5/1f/*Enterobacter cloacae*) were derived from the Collection of Microorganisms of Department of Biotechnology and Food Microbiology, Wroclaw University of Environmental and Life Sciences. Bacterial hosts of these phages are environmental isolates also deposited in the same collection. The basic characteristics of bacteriophages used in the studies are presented in Table S12.

### 3.4. Amplification of Bacteriophages

Bacteriophages were amplified with a two-stage method described previously by Skaradzińska et al. [44] with some modifications. Briefly, a single colony of the host strain was transferred to 30 mL of liquid Luria-Bertani (LB) medium (10 g/L bacto-tryptone, 5 g/L bacto-yeast extract, 10 g/L NaCl) medium and incubated for 20 h (37 °C, 150 rpm). After incubation, 10 mL of respective bacteriophage preparation was added, and the phage-bacteria culture was incubated for the next 24 h in the same conditions. Then, the culture was centrifuged (2000× *g*, 5 min) and filtered through syringe filters with a diameter of 33 mm and polyethersulfone (PES) membrane pore size of 0.22 μm (Merck-Millipore, Burlington, MA, USA). The filtrate was added to a 20 h host bacterial culture prepared analogously as at the previous stage and subsequently incubated for 24 h (37 °C, 150 rpm). The culture was again centrifuged (2000× *g*, 5 min) and filtered through syringe filters with a diameter of 33 mm and PES membrane pore size of 0.22 μm (Merck-Millipore, Burlington, MA, USA). Phage titer in preparations was determined using the standard routine test dilution (RTD) and double-layer method [45]. All phage preparations used in the experiments had a phage titer brought to a value of 5 × 10^7^ pfu/mL.

### 3.5. Influence of RLs on the Activity of Bacteriophages

One hundred microliters of bacteriophage preparation with a previously determined phage titer were introduced into a plastic tube, and 100 µL of RLs were added to obtain final concentrations: 500 mg/L, 250 mg/L, and 125 mg/L. The final volume of the solutions was 200 µL. The mixtures were incubated for 24 h at 37 °C, and then phage titer was determined with a standard double-layer method [45].

### 3.6. Bacterial Control Lysates

Bacterial control lysates, along with phage lysates, were prepared according to the procedure described by Kurzepa-Skaradzinska et al. [46] with some modifications. Two bacterial cultures were prepared by transferring two single colonies into each of the two plastic tubes containing 30 mL of LB. The cultures were incubated for 20 h (37 °C, 150 rpm), and after incubation, 10 mL of respective bacteriophage preparation was added to one culture. Optical density at 600 nm (OD_600_) of the culture with phage was monitored in 30 min intervals, and when it started to drop (amplified phages were released from cells due to bacterial lysis), the other culture was disrupted by ultrasounds with an amplitude of 40 for 10 min (Vibra cell, Sonics and Materials, East Lyme, CT, USA). After ultrasounds, 100 μL of lysozyme was added to the disrupted sample, which was then incubated on ice for 30 min before being stored at −80 °C for a minimum of 24 h. Both cultures were filtered through syringe filters with a diameter of 33 mm and PES membrane pore size of 0.22 μm (Merck-Millipore, USA).

### 3.7. Effect of Rhamnolipids and Bacteriophages on the Growth of Candida albicans

The experiment examining the effects of RLs and bacteriophages on *C. albicans* was carried out in liquid culture using a 96-well plate. Each well initially contained 100 µL of YPD medium. To achieve the desired concentrations (200 mg/L, 100 mg/L, and 50 mg/L for RLs and 5 × 10^7^ pfu/mL for phages), 100 µL of RLs or bacteriophages prepared at twice higher concentrations were added. A control well with 200 µL of YPD medium was included. Each well was inoculated with 1 µL of an overnight culture of *C. albicans,* and the plate was incubated for 24 h at 37 °C. Optical density at 600 nm (OD_600_) was measured using a microplate reader TECAN Spark 10 M (Tecan Group Ltd., Männedorf, Switzerland). Each variant was tested in triplicate, and then the results were averaged and presented in a graph. A Student’s *t*-test was performed to test the significance of the differences between the control and test samples.

### 3.8. Anti-Adhesion Assays

Inhibition of microbial adhesion by RLs, bacteriophages, and their combinations was tested in 96-well plates (Sarstedt, Nümbrecht, Germany). Briefly, the wells of a sterile 96-well flat-bottom plate were filled with 100 μL of 50, 100, and 200 mg/L RLs, bacteriophages (5 × 10^7^ pfu/mL), and their mixtures. The plates were incubated for 2 h at 37 °C on a rotary shaker (MixMate, Eppendorf, Hamburg, Germany) at 300 rpm and subsequently washed twice with sterile water. Control wells contained YPD medium only. The overnight culture of the *Candida* strain was centrifuged, washed twice with sterile water, and re-suspended in YPD medium to an optical density OD_600_ = 0.6. A 100 μL aliquot of *Candida* suspension was added and incubated in the wells. After a 2 h incubation at 37 °C in a rotary shaker at 300 rpm, nonadherent cells were removed by three washes with sterile water. Then, the plates were stained with 0.1% crystal violet for 5 min and again washed three times with sterile water. The adherent *Candida* cells were permeabilized, and the dye was resolubilized with 100 µL of isopropanol per well. Crystal violet optical density readings of each well were taken at 570 nm on the microplate reader TECAN Spark 10 M (Tecan Group Ltd., Männedorf, Switzerland). The assays were carried out in three replicates.

### 3.9. In Vitro Anti-Biofilm Assay

Briefly, 96-well plates were seeded with 100 µL aliquots of *C. albicans* cells (1.0 × 10^6^ cells/mL) in YPD medium and incubated statically at 37 °C for 1.5 h for initial adhesion. After the adhesion of cells to the plate surface, the suspension was gently aspirated and washed with sterile water to remove non-adhering cells. Fresh YPD medium with different concentrations of RLs (50, 100, and 200 mg/L), bacteriophages (5 × 10^7^ pfu/mL), and their mixtures were added, followed by incubation for another 24 h. After incubation, the supernatant was gently aspirated, and each well was washed with sterile water. Then, the plates were stained with 0.1% crystal violet for 5 min and again washed three times with sterile water. The adherent *Candida* cells were permeabilized, and the dye was resolubilized with 100 µL of isopropanol per well. Crystal violet optical density readings of each well were taken at 570 nm on the microplate reader TECAN Spark 10 M (Tecan Group Ltd., Männedorf, Switzerland). The assays were carried out in three replicates.

### 3.10. Quantification of Gene Expression by Quantitative Real-Time PCR (qRT-PCR)

The effect of RLs (200 mg/L) and RLs with bacteriophages (5 × 10^7^ pfu/mL) on the expression of hypha-specific genes *HWP1* (hyphal wall protein 1), *ALS3* (agglutinin-like sequence 3), *ECE1* (extent of cell elongation 1), and *SAP4* (secreted aspartyl protease 4) was evaluated by two-step quantitative real-time polymerase chain reaction (qRT-PCR). Samples contained *C. albicans ATCC 10231* (GenBank genome assembly number GCA_015227795.1) (5.0 × 10^6^ cells/mL) culture, and RLs (200 mg/L) or RLs with bacteriophages (5 × 10^7^ pfu/mL) were incubated at 37 °C for 6 h and then centrifuged at 1000 × *g* for 5 min. Subsequently, the RNA was extracted from cells using a Total RNA Mini Plus kit (A&A Biotechnology, Gdańsk, Poland). Each sample was treated with DNase I (Thermo Scientific, Waltham, MA, USA) for 30 min at 37 °C, and then with EDTA for 10 min at 65 °C. The RNA quantities were measured using a Biochrom WPA Biowave II spectrophotometer (Biochrom Ltd., Cambridge, UK) equipped with a TrayCell system (Hellma Analytics, Müllheim, Germany). The cDNA synthesis was conducted according to Maxima First Strand cDNA. Synthesis kits for qRT-PCR (Thermo Scientific, Waltham, MA, USA) were used according to the manufacturer’s instructions. The qRT-PCR analyses were carried out using a DyNAmo Flash SYBR Green qPCR kit (Thermo Scientific) and the Eco Real-Time PCR System (Illumina, San Diego, CA, USA) with 1 ng/µL cDNA in the final reaction. Sequences of all primers (*ALS3*, *ECE1*, *HWP1*, and *SAP4*) used in the study were taken from publication [47] and are listed in Table S13. The results were normalized to the actin gene (*ACT1*) and analyzed using the ddCT method [48]. The experiment was performed in three biological replicates. Statistical significance was determined using binomial, unpaired Student’s *t*-test. A *p*-value of 0.05 was considered to be statistically significant.

### 3.11. Hypha Formation

Hyphal growth assay was performed in 10 mL of RPMI-1640 medium (Sigma-Aldrich, St. Louis, MO, USA). Cell suspension was diluted at 1 × 10^7^ cells/mL in medium and incubated with RLs (200 mg/L) and RLs with bacteriophages (5 × 10^7^ pfu/mL) at 37 °C for 5 h. Aliquots of samples were visualized under a bright field using a 40 objective lens by Zeiss AXIO Scope A1 microscope (Zeiss, Oberkochen, Germany) and photographed.

### 3.12. Statistical Analysis

Data were reported as mean values with the standard deviations of the mean. The analysis of variance (ANOVA) followed by least significant difference (LSD) post-hoctest was performed using STATISTICA ver. 13.3 (TIBCO). Statistical significance was considered at a significance level of *p* < 0.05

## 4. Conclusions

Eliminating biofilms remains a formidable challenge due to their complex structure and resistance mechanisms. Traditional antifungal treatments often struggle to penetrate the dense biofilm matrix, resulting in incomplete eradication. Developing strategies to disrupt biofilm formation is therefore a crucial step in combating persistent infections caused by biofilm-forming microorganisms such as *Candida*.

Recent approaches have focused on preventing biofilm formation by developing anti-adhesive surfaces or inhibiting pathogen adhesion. In this study, we demonstrated that the combination of RLs and bacteriophages consistently exhibited a stronger inhibitory effect on *Candida* biofilm formation than either agent alone. Specifically, the mixture of 200 mg/L RLs and phage BF9 resulted in a 94.8% reduction compared to the untreated control. Similarly, in an experimental model where RLs and bacteriophages were introduced concurrently with *C. albicans* cells, a synergistic effect was observed across all biosurfactant concentrations and phages tested. The most pronounced inhibition occurred with 200 mg/L RLs combined with phage LO5/1f, achieving nearly complete biofilm suppression (~96% reduction vs. control). Notably, this effect was further supported by a decreased expression of biofilm-associated genes in *Candida* when RLs and phages were applied together, compared to individual treatments.

Although the molecular mechanisms underlying this phenomenon remain unclear, our findings highlight a potent synergistic interaction between RLs and phages, opening new avenues for the development of innovative anti-biofilm strategies.

## Figures and Tables

**Table 1:** Bacteriophage titer (pfu/mL) after incubation with RLs solutions. The same subscript letter designates homogenous groups within the same bacteriophage at a *p*-value of <0.05.

Bacteriophage	Phage Titer [pfu/mL]	Phage Titer After Incubation with 125 mg/L RLs	Phage Titer After Incubation with 250 mg/L RLs	Phage Titer After Incubation with 500 mg/L RLs
BF9	7.51 ± 5.20 × 10^8^ _a_	1.55 ± 0.13 × 10^8^ _b_	1.38 ± 0.16 × 10^8^ _c_	1.23 ± 0.35 × 10^8^ _c_
BF15	2.35 ± 0.62 × 10^9^ _a_	1.91 ± 0.14 × 10^9^ _ab_	1.68 ± 0.15 × 10^9^ _ab_	1.43 ± 0.23 × 10^9^ _c_
BF17	1.70 ± 0.63 × 10^9^ _a_	9.51 ± 0.77 × 10^8^ _b_	1.60 ± 0.35 × 10^9^ _ab_	1.15 ± 0.19 × 10^9^ _a_
FD	2.50 ± 1.39 × 10^10^ _a_	1.35 ± 0.32 × 10^10^ _a_	9.50 ± 1.02 × 10^9^ _ab_	5.00 ± 4.44 × 10^9^ _b_
Felix	9.13 ± 1.00 × 10^9^ _a_	9.10 ± 0.44 × 10^9^ _a_	8.02 ± 0.38 × 10^9^ _a_	8.34 ± 0.23 × 10^9^ _a_
JG004	1.97 ± 1.08 × 10^9^ _a_	1.02 ± 0.06 × 10^9^ _ab_	9.50 ± 1.39 × 10^8^ _ab_	8.25 ± 0.77 × 10^8^ _b_
LO5/1f	1.92 ± 1.71 × 10^9^ _a_	2.55 ± 1.36 × 10^8^ _ab_	2.75 ± 0.95 × 10^8^ _ab_	2.00 ± 1.74 × 10^8^ _b_
T4	3.50 ± 1.30 × 10^8^ _a_	3.31 ± 1.00 × 10^8^ _a_	2.80 ± 0.42 × 10^8^ _a_	2.00 ± 0.35 × 10^8^ _a_
TO1/6f	5.10 ± 3.72 × 10^9^ _a_	2.05 ± 1.06 × 10^9^ _ab_	1.26 ± 0.32 × 10^9^ _ab_	8.35 ± 1.75 × 10^8^ _b_
TO1/7f	4.97 ± 2.30 × 10^9^ _a_	4.49 ± 1.25 × 10^9^ _a_	3.06 ± 0.48 × 10^9^ _ab_	2.02 ± 1.11 × 10^9^ _b_

## Data Availability

The datasets generated during and/or analyzed during the current study are available from the corresponding author upon reasonable request.

## Supplementary Materials

The following supporting information can be downloaded at: https://www.mdpi.com/article/10.3390/molecules30081772/s1, Table S1: One-way ANOVA and least significant difference (LSD) post-hoc comparisons of the means of bacteriophage titer (pfu/mL) after incubation with RLs solutions; Figure S1: Effect of rhamnolipids and bacteriophages on the growth of *Candida albicans*; Table S2: One-way ANOVA and least significance difference (LSD) post-hoc comparisons of means of optical density (OD 600) of *C. albicans* treated with RLs and bacteriophages; Table S3: The percentage of biofilm formation by *C. albicans* on surfaces pretreated with RLs, bacteriophages, and their combinations; Table S4: One-way ANOVA and least significance difference (LSD) post-hoc comparisons of means of percentage of biofilm formation by *C. albicans* on surfaces pretreated with RLs, bacteriophages, and their combinations. Data are presented as *p*-values between samples for each phage; Table S5: One-way ANOVA and least significance difference (LSD) post-hoc comparisons of means of percentage of biofilm formation by *C. albicans* on surfaces pretreated with RLs, bacteriophages, and their combinations. Data are presented as *p*-values between phages for each sample; Table S6: The percentage of biofilm formation by *C. albicans* in the presence of filtered culture of host bacteria. The results represent the averages of triplicate experiments; Table S7: The percentage of biofilm formation by *C. albicans* in the presence of RLs, bacteriophages, and their combinations; Table S8: One-way ANOVA and least significance difference (LSD) post-hoc comparisons of means of percentage of biofilm formation by *C. albicans* in the presence of RLs, bacteriophages, and their combinations. Data are presented as *p*-values between samples for each phage; Table S9: One-way ANOVA and least significance difference (LSD) post-hoc comparisons of means of percentage of biofilm formation by *C. albicans* in the presence of RLs, bacteriophages, and their combinations. Data are presented as *p*-values between phages for each sample; Table S10: One-way ANOVA and least significance difference (LSD) post-hoc comparisons of means of expression of genes responsible for biofilm formation in *Candida* yeast treated with RLs, phages, and their combinations. Data are presented as *p*-values between samples for each phage; Table S11: Chemical structure and relative abundance of RLs used in this work; Table S12: Characteristics of bacteriophages used in the studies; Table S13: List of primers used for qRT-PCR experiments. References [49,50,51,52,53,54] are cited in Supplementary Materials.
