# Supplementary material for: Bacteriophages Improve the Effectiveness of Rhamnolipids in Combating the Biofilm of Candida albicans
Source: Molecules. 2025 Apr 15;30(8):1772. doi: 10.3390/molecules30081772 (PMC12029421; doi:10.3390/molecules30081772)
Supplement: Supplementary file 1 [file molecules-30-01772-s001.zip › molecules-3515028-supplementary.pdf]

Supplementary materials

Table S1. One-way ANOVA and least significant difference (LSD) post-hoc comparisons of the means of bacteriophage titer (pfu/mL) after incubation with RLs solutions. Data are presented as P-values between samples. Values depicted in red are statistically significant at a 95% confidence interval

BF9

	Phage titer	125 mg	250 mg	500 mg
Phage titer		0,000052	0,031132	0,017935
125 MG	0,000052		0,000825	0,001288
250 MG	0,031132	0,000825		0,729826
500 MG	0,017935	0,001288	0,729826	

BF15

	Phage titer	125 mg	250 mg	500 mg
Phage titer		0,212640	0,050010	0,007995
125 MG	0,212640		0,369185	0,063494
250 MG	0,050010	0,369185		0,263996
500 MG	0,007995	0,063494	0,263996	

BF17

	Phage titer	125 mg	250 mg	500 mg
Phage titer		0,037618	0,917588	0,142179
125 MG	0,037618		0,044436	0,414617
250 MG	0,917588	0,044436		0,166703
500 MG	0,142179	0,414617	0,166703	

FD

	Phage titer	125 mg	250 mg	500 mg
Phage titer		0,352203	0,137055	0,007982
125 MG	0,352203		0,524968	0,035779

JG004

	Phage titer	125 mg	250 mg	500 mg
Phage titer		0,105282	0,069564	0,033196
125 mg/L	0,105282		0,795340	0,478911
250 mg/L	0,069564	0,795340		0,647828
500 mg/L	0,033196	0,478911	0,647828	

LO5/1f

	Phage titer	125 mg	250 mg	500 mg
Phage titer		0,055615	0,077205	0,025240
125 mg	0,055615		0,838374	0,625475
250 mg	0,077205	0,838374		0,493042
500 mg	0,025240	0,625475	0,493042	

T4

	Phage titer	125 mg	250 mg	500 mg
Phage titer		0,892532	0,497193	0,068628
125 mg	0,892532		0,583228	0,085205
250 mg	0,497193	0,583228		0,201477
500 mg	0,068628	0,085205	0,201477	

TO1/6f

	Phage titer	125 mg	250 mg	500 mg
Phage titer		0,296199	0,115537	0,039977
125 mg	0,296199		0,535375	0,219616

250 MG	0,137055	0,524968		0,100564	250 mg	0,115537	0,535375		0,513176
500 MG	0,007982	0,035779	0,100564		500 mg	0,039977	0,219616	0,513176	

**FELIX**					**TO1/7f**				
	Phage titer	125 mg	250 mg	500 mg		Phage titer	125 mg	250 mg	500 mg
Phage titer		0,999174	0,050254	0,152736	Phage titer		0,930488	0,336082	0,042268
125 MG	0,999174		0,050170	0,152493	125 mg	0,930488		0,377950	0,048644
250 MG	0,050254	0,050170		0,490479	250 mg	0,336082	0,377950		0,201903
500 MG	0,152736	0,152493	0,490479		500 mg	0,042268	0,048644	0,201903	

Figure S1. Effect of rhamnolipids and bacteriophages on the growth of *Candida albicans*. Each variant was carried out in triplicate. The same letters designate homogenous groups comparing genes expression within the same sample at a P-value of <0.05.

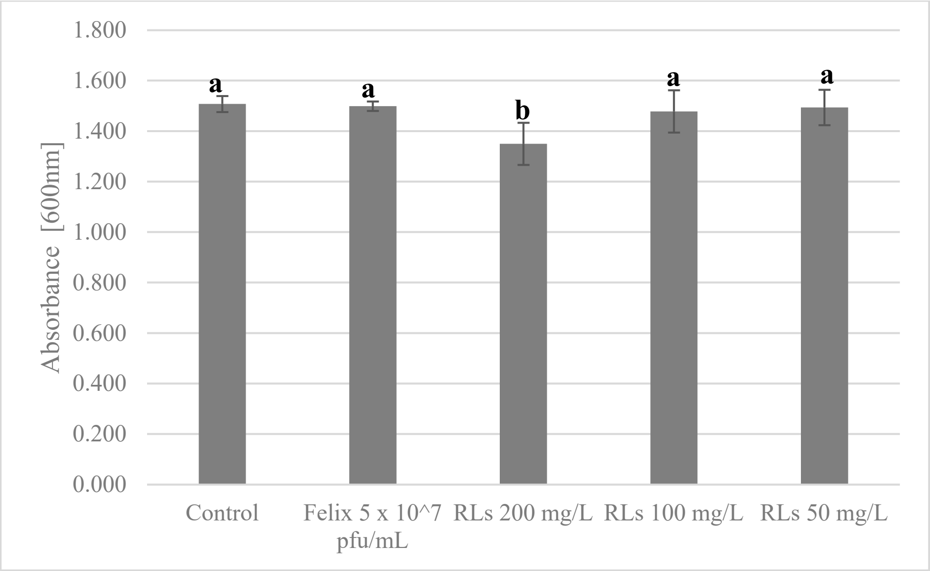

Table S2. One-way ANOVA and least significance difference (LSD) post-hoc comparisons of means of optical density (OD 600) of *C. albicans* treated with RLs and bacteriophages. Data are presented as P-values between samples. Values depicted in red are statistically significant at a 95% confidence interval.

	CONTROL	FELIX 5 X 10^7 PFU/ML	RLS 200 MG/L	RLS 100 MG/L	RLS 50 MG/L
**CONTROL**		0,870990	0,012651	0,585216	0,798079
**FELIX 5 X 10^7 PFU/ML**	0,870990		0,016824	0,699476	0,925319
**RLS 200 MG/L**	0,012651	0,016824		0,033261	0,019840
**RLS 100 MG/L**	0,585216	0,699476	0,033261		0,769433
**RLS 50 MG/L**	0,798079	0,925319	0,019840	0,769433	

Table S3. The percentage of biofilm formation by *C. albicans* on surfaces **pretreated** with RLs, bacteriophages, and their combinations. The results represent the averages of triplicate experiments ± SD. The same uppercase letters designate homogenous groups comparing bacteriophages within the same treatment, and the same lowercase letters designate homogenous groups comparing control and samples within the same bacteriophages at a P-value of <0.05

Phage	BF17	Felix	JG004	BF15	FD	TO1/7f	TO1/6f	LO5/1f	BF9	T4
Control	**100.0** $^{A}_{a}$									
RLs 50 mg/L	**23.7** ± 3.3 $^{A}_{b}$									
Bacteriophage + RLs 50 mg/L	**19.5** ± 3.0 $^{AB}_{c}$	**20.7** ± 2.1 $^{AB}_{c}$	**19.7** ± 1.5 $^{B}_{c}$	**22.2** ± 2.5 $^{D}_{b}$	**21.5** ± 0.9 $^{D}_{c}$	**18.5** ± 4.8 $^{AC}_{c}$	**17.1** ± 4.6$^{E}_{c}$	**17.8**± 4.1 $^{CE}_{c}$	**16.8** ± 5.1 $^{E}_{c}$	**15.0** ± 5.9 $^{F}_{c}$
RLs 100 mg/L	**15.0** ± 3.7 $^{A}_{d}$	**15.0** ± 3.7 $^{A}_{d}$	**15.0** ± 3.7 $^{A}_{d}$	**15.0** ± 3.7 $^{A}_{c}$	**15.0** ± 3.7 $^{A}_{d}$	**15.0** ± 3.7 $^{A}_{d}$	**15.0** ± 3.7 $^{A}_{d}$	**15.0** ± 3.7 $^{A}_{d}$	**15.0** ± 3.7 $^{A}_{c}$	**15.0** ± 3.7 $^{A}_{c}$
Bacteriophage + RLs 100 mg/L	**13.3** ± 6.1 $^{A}_{d}$	**14.3** ± 0.7 $^{F}_{d}$	**11.3** ± 2.0 $^{DE}_{e}$	**14.5** ± 1.4 $^{F}_{c}$	**12.6** ± 3.3 $^{AB}_{e}$	**11.3** ± 0.6 $^{DE}_{e}$	**12.2** ± 5.1 $^{BC}_{e}$	**12.2** ± 2.9 $^{BC}_{e}$	**11.8** ± 4.1 $^{CD}_{d}$	**10.6** ± 3.2 $^{E}_{d}$
RLs 200 mg/L	**9.9** ± 4.9 $^{A}_{e}$	**9.9** ± 4.9 $^{A}_{e}$	**9.9** ± 4.9 $^{A}_{e}$	**9.9** ± 4.9 $^{A}_{d}$	**9.9** ± 4.9 $^{A}_{f}$	**9.9** ± 4.9 $^{A}_{e}$	**9.9** ± 4.9 $^{A}_{f}$	**9.9** ± 4.9 $^{A}_{f}$	**9.9** ± 4.9 $^{A}_{d}$	**9.9** ± 4.9 $^{A}_{d}$
Bacteriophage + RLs 200 mg/L	**8.5** ± 6.7 $^{A}_{e}$	**8.1** ± 2.8 $^{A}_{e}$	**5.3** ± 6.7 $^{B}_{f}$	**5.5** ± 8.5 $^{B}_{e}$	**7.1** ± 7.3 $^{DE}_{g}$	**6.6** ± 2.5 $^{CD}_{f}$	**6.2** ± 2.2 $^{C}_{g}$	**7.4** ± 4.5 $^{E}_{g}$	**5.2** ± 6.0 $^{B}_{e}$	**5.4** ± 1.4 $^{B}_{e}$
Bacteriophage (5x10^{7} pfu/mL)	**93.7** ± 1.7 $^{AD}_{f}$	**90.8** ± 1.8 $^{AB}_{f}$	**86.0** ± 4.1 $^{C}_{g}$	**87.0** ± 0.6 $^{CE}_{f}$	**89.3** ± 1.3 $^{BE}_{h}$	**91.3** ± 1.0 $^{AB}_{g}$	**80.4** ± 1.0 $^{F}_{h}$	**95.1** ± 1.5 $^{D}_{h}$	**85.4** ± 2.1 $^{C}_{f}$	**82.3** ± 2.0 $^{F}_{f}$

Table S4. One-way ANOVA and least significance difference (LSD) post-hoc comparisons of means of percentage of biofilm formation by *C. albicans* on surfaces **pretreated** with RLs, bacteriophages, and their combinations. Data are presented as P-values between samples for each phage. Values depicted in red are statistically significant at a 95% confidence interval.

Phage	Sample	Control	RLs 50 mg/L	RLs 100 mg/L	RLs 200 mg/L	BF + RLs 50 mg/L	BF + RLs 100 mg/L	BF + RLs 200 mg/L	Phage
BF17	Control		0,000000	0,000000	0,000000	0,000000	0,000000	0,000000	0,000007
	RLs 50 mg/L	0,000000		0,000000	0,000000	0,000479	0,000000	0,000000	0,000000
	RLs 100 mg/L	0,000000	0,000000		0,000072	0,000287	0,093704	0,000005	0,000000
	RLs 200 mg/L	0,000000	0,000000	0,000072		0,000000	0,002866	0,168319	0,000000
	BF + RLs 50 mg/L	0,000000	0,000479	0,000287	0,000000		0,000009	0,000000	0,000000
	BF + RLs 100 mg/L	0,000000	0,000000	0,093704	0,002866	0,000009		0,000142	0,000000
	BF + RLs 200 mg/L	0,000000	0,000000	0,000005	0,168319	0,000000	0,000142		0,000000
	Phage	0,000007	0,000000	0,000000	0,000000	0,000000	0,000000	0,000000	
Felix	control		0,000000	0,000000	0,000000	0,000000	0,000000	0,000000	0,000000
	RLs 50 mg/L	0,000000		0,000000	0,000000	0,000424	0,000000	0,000000	0,000000
	RLs 100 mg/L	0,000000	0,000000		0,000063	0,000253	0,468791	0,000004	0,000000
	RLs 200 mg/L	0,000000	0,000000	0,000063		0,000000	0,000280	0,163012	0,000000
	BF + RLs 50 mg/L	0,000000	0,000424	0,000253	0,000000		0,000057	0,000000	0,000000
	BF + RLs 100 mg/L	0,000000	0,000000	0,468791	0,000280	0,000057		0,000016	0,000000
	BF + RLs 200 mg/L	0,000000	0,000000	0,000004	0,163012	0,000000	0,000016		0,000000
	Phage	0,000000	0,000000	0,000000	0,000000	0,000000	0,000000	0,000000	
JG004	control		0,000000	0,000000	0,000000	0,000000	0,000000	0,000000	0,000000
	RLs 50 mg/L	0,000000		0,000005	0,000000	0,007725	0,000000	0,000000	0,000000

	RLs 100 mg/L	0,000000	0,000005		0,001192	0,002300	0,010864	0,000001	0,000000
	RLs 200 mg/L	0,000000	0,000000	0,001192		0,000001	0,308942	0,002460	0,000000
	BF + RLs 50 mg/L	0,000000	0,007725	0,002300	0,000001		0,000007	0,000000	0,000000
	BF + RLs 100 mg/L	0,000000	0,000000	0,010864	0,308942	0,000007		0,000273	0,000000
	BF + RLs 200 mg/L	0,000000	0,000000	0,000001	0,002460	0,000000	0,000273		0,000000
	Phage	0,000000	0,000000	0,000000	0,000000	0,000000	0,000000	0,000000	
	control		0,000000	0,000000	0,000000	0,000000	0,000000	0,000000	0,000000
	RLs 50 mg/L	0,000000		0,000000	0,000000	0,088129	0,000000	0,000000	0,000000
	RLs 100 mg/L	0,000000	0,000000		0,000014	0,000000	0,545356	0,000000	0,000000
	RLs 200 mg/L	0,000000	0,000000	0,000014		0,000000	0,000046	0,000072	0,000000
BF15	BF + RLs 50 mg/L	0,000000	0,088129	0,000000	0,000000		0,000000	0,000000	0,000000
	BF + RLs 100 mg/L	0,000000	0,000000	0,545356	0,000046	0,000000		0,000000	0,000000
	BF + RLs 200 mg/L	0,000000	0,000000	0,000000	0,000072	0,000000	0,000000		0,000000
	Phage	0,000000	0,000000	0,000000	0,000000	0,000000	0,000000	0,000000	
	control		0,000000	0,000000	0,000000	0,000000	0,000000	0,000000	0,000000
	RLs 50 mg/L	0,000000		0,000000	0,000000	0,024964	0,000000	0,000000	0,000000
FD	RLs 100 mg/L	0,000000	0,000000		0,000026	0,000002	0,014891	0,000000	0,000000
	RLs 200 mg/L	0,000000	0,000000	0,000026		0,000000	0,006953	0,004910	0,000000
	BF + RLs 50 mg/L	0,000000	0,024964	0,000002	0,000000		0,000000	0,000000	0,000000
	BF + RLs 100 mg/L	0,000000	0,000000	0,014891	0,006953	0,000000		0,000010	0,000000
	BF + RLs 200 mg/L	0,000000	0,000000	0,000000	0,004910	0,000000	0,000010		0,000000
	Phage	0,000000	0,000000	0,000000	0,000000	0,000000	0,000000	0,000000	
TO1/7f	control		0,000000	0,000000	0,000000	0,000000	0,000000	0,000000	0,000000

	RLs 50 mg/L	0,000000		0,000000	0,000000	0,000018	0,000000	0,000000	0,000000
	RLs 100 mg/L	0,000000	0,000000		0,000023	0,001134	0,000524	0,000000	0,000000
	RLs 200 mg/L	0,000000	0,000000	0,000023		0,000000	0,137344	0,001676	0,000000
	BF + RLs 50 mg/L	0,000000	0,000018	0,001134	0,000000		0,000000	0,000000	0,000000
	BF + RLs 100 mg/L	0,000000	0,000000	0,000524	0,137344	0,000000		0,000067	0,000000
	BF + RLs 200 mg/L	0,000000	0,000000	0,000000	0,001676	0,000000	0,000067		0,000000
	Phage	0,000000	0,000000	0,000000	0,000000	0,000000	0,000000	0,000000	
TO1/F6	control		0,000000	0,000000	0,000000	0,000000	0,000000	0,000000	0,000000
	RLs 50 mg/L	0,000000		0,000000	0,000000	0,000001	0,000000	0,000000	0,000000
	RLs 100 mg/L	0,000000	0,000000		0,000024	0,028309	0,004695	0,000000	0,000000
	RLs 200 mg/L	0,000000	0,000000	0,000024		0,000000	0,019748	0,000576	0,000000
	BF + RLs 50 mg/L	0,000000	0,000001	0,028309	0,000000		0,000033	0,000000	0,000000
	BF + RLs 100 mg/L	0,000000	0,000000	0,004695	0,019748	0,000033		0,000004	0,000000
	BF + RLs 200 mg/L	0,000000	0,000000	0,000000	0,000576	0,000000	0,000004		0,000000
	Phage	0,000000	0,000000	0,000000	0,000000	0,000000	0,000000	0,000000	
LO5/1f	control		0,000000	0,000000	0,000000	0,000000	0,000000	0,000000	0,000070
	RLs 50 mg/L	0,000000		0,000000	0,000000	0,000010	0,000000	0,000000	0,000000
	RLs 100 mg/L	0,000000	0,000000		0,000045	0,007923	0,007559	0,000000	0,000000
	RLs 200 mg/L	0,000000	0,000000	0,000045		0,000000	0,024588	0,015218	0,000000
	BF + RLs 50 mg/L	0,000000	0,000010	0,007923	0,000000		0,000016	0,000000	0,000000
	BF + RLs 100 mg/L	0,000000	0,000000	0,007559	0,024588	0,000016		0,000088	0,000000
	BF + RLs 200 mg/L	0,000000	0,000000	0,000000	0,015218	0,000000	0,000088		0,000000
	Phage	0,000070	0,000000	0,000000	0,000000	0,000000	0,000000	0,000000	

BF9	control		0,000000	0,000000	0,000000	0,000000	0,000000	0,000000	0,000000
	RLs 50 mg/L	0,000000		0,000000	0,000000	0,000003	0,000000	0,000000	0,000000
	RLs 100 mg/L	0,000000	0,000000		0,000090	0,086014	0,005100	0,000000	0,000000
	RLs 200 mg/L	0,000000	0,000000	0,000090		0,000003	0,069496	0,000208	0,000000
	BF + RLs 50 mg/L	0,000000	0,000003	0,086014	0,000003		0,000113	0,000000	0,000000
	BF + RLs 100 mg/L	0,000000	0,000000	0,005100	0,069496	0,000113		0,000005	0,000000
	BF + RLs 200 mg/L	0,000000	0,000000	0,000000	0,000208	0,000000	0,000005		0,000000
	Phage	0,000000	0,000000	0,000000	0,000000	0,000000	0,000000	0,000000	
T4	control		0,000000	0,000000	0,000000	0,000000	0,000000	0,000000	0,000000
	RLs 50 mg/L	0,000000		0,000000	0,000000	0,000000	0,000000	0,000000	0,000000
	RLs 100 mg/L	0,000000	0,000000		0,000072	0,945069	0,000314	0,000000	0,000000
	RLs 200 mg/L	0,000000	0,000000	0,000072		0,000083	0,477841	0,000252	0,000000
	BF + RLs 50 mg/L	0,000000	0,000000	0,945069	0,000083		0,000363	0,000000	0,000000
	BF + RLs 100 mg/L	0,000000	0,000000	0,000314	0,477841	0,000363		0,000058	0,000000
	BF + RLs 200 mg/L	0,000000	0,000000	0,000000	0,000252	0,000000	0,000058		0,000000
	Phage	0,000000	0,000000	0,000000	0,000000	0,000000	0,000000	0,000000	

Table S5. One-way ANOVA and least significance difference (LSD) post-hoc comparisons of means of percentage of biofilm formation by *C. albicans* on surfaces **pretreated** with RLs, bacteriophages, and their combinations. Data are presented as P-values between phage for each sample. Values depicted in red are statistically significant at a 95% confidence interval.

Sample	Phage	BF17	Felix	JG004	BF15	FD	TO1/7f	TO1/6f	LO5/1f	BF9	T4
BF + RLs 50 mg/L	BF17		1,000000	0,650419	0,000090	0,001450	0,080608	0,000389	0,007305	0,000112	0,000000
	Felix	1,000000		0,650419	0,000090	0,001450	0,080608	0,000389	0,007305	0,000112	0,000000
	JG004	0,650419	0,650419		0,000262	0,004199	0,032320	0,000133	0,002556	0,000039	0,000000
	BF15	0,000090	0,000090	0,000262		0,247038	0,000002	0,000000	0,000000	0,000000	0,000000
	FD	0,001450	0,001450	0,004199	0,247038		0,000021	0,000000	0,000002	0,000000	0,000000
	TO1/7f	0,080608	0,080608	0,032320	0,000002	0,000021		0,025535	0,265520	0,007948	0,000004
	TO1/6f	0,000389	0,000389	0,000133	0,000000	0,000000	0,025535		0,219510	0,598414	0,000857
	LO5/1f	0,007305	0,007305	0,002556	0,000000	0,000002	0,265520	0,219510		0,086514	0,000045
	BF9	0,000112	0,000112	0,000039	0,000000	0,000000	0,007948	0,598414	0,086514		0,002974
	T4	0,000000	0,000000	0,000000	0,000000	0,000000	0,000004	0,000857	0,000045	0,002974	
BF + RLs 100 mg/L	BF17		0,008419	0,000010	0,002378	0,065540	0,000010	0,003725	0,004734	0,000386	0,000000
	Felix	0,008419		0,000000	0,585358	0,000093	0,000000	0,000005	0,000006	0,000001	0,000000
	JG004	0,000010	0,000000		0,000000	0,000896	0,981106	0,018556	0,014782	0,127817	0,069448
	BF15	0,002378	0,585358	0,000000		0,000026	0,000000	0,000001	0,000002	0,000000	0,000000
	FD	0,065540	0,000093	0,000896	0,000026		0,000847	0,197231	0,233344	0,031810	0,000011
	TO1/7f	0,000010	0,000000	0,981106	0,000000	0,000847		0,017621	0,014028	0,122487	0,072721
	TO1/6f	0,003725	0,000005	0,018556	0,000001	0,197231	0,017621		0,917490	0,341598	0,000229
	LO5/1f	0,004734	0,000006	0,014782	0,000002	0,233344	0,014028	0,917490		0,293384	0,000179

	BF9	0,000386	0,000001	0,127817	0,000000	0,031810	0,122487	0,341598	0,293384		0,002218
	T4	0,000000	0,000000	0,069448	0,000000	0,000011	0,072721	0,000229	0,000179	0,002218	
BF + RLs 200 mg/L	BF17		1,000000	0,000000	0,000000	0,000173	0,000009	0,000000	0,002277	0,000000	0,000000
	Felix	1,000000		0,000000	0,000000	0,000173	0,000009	0,000000	0,002277	0,000000	0,000000
	JG004	0,000000	0,000000		0,434963	0,000016	0,000305	0,008042	0,000001	0,910516	0,642362
	BF15	0,000000	0,000000	0,434963		0,000098	0,001958	0,044299	0,000008	0,373376	0,748409
	FD	0,000173	0,000173	0,000016	0,000098		0,213637	0,013804	0,281993	0,000012	0,000046
	TO1/7f	0,000009	0,000009	0,000305	0,001958	0,213637		0,172565	0,026796	0,000234	0,000918
	TO1/6f	0,000000	0,000000	0,008042	0,044299	0,013804	0,172565		0,001110	0,006224	0,022563
	LO5/1f	0,002277	0,002277	0,000001	0,000008	0,281993	0,026796	0,001110		0,000001	0,000004
	BF9	0,000000	0,000000	0,910516	0,373376	0,000012	0,000234	0,006224	0,000001		0,564856
	T4	0,000000	0,000000	0,642362	0,748409	0,000046	0,000918	0,022563	0,000004	0,564856	
BF	BF17		0,050602	0,000018	0,000098	0,004608	0,094103	0,000000	0,321941	0,000008	0,000000
	Felix	0,050602		0,002215	0,011925	0,280458	0,750571	0,000000	0,005702	0,000942	0,000006
	JG004	0,000018	0,002215		0,466690	0,026331	0,001046	0,000664	0,000002	0,717274	0,016956
	BF15	0,000098	0,011925	0,466690		0,113233	0,005799	0,000118	0,000010	0,280458	0,003213
	FD	0,004608	0,280458	0,026331	0,113233		0,167698	0,000003	0,000436	0,011925	0,000068
	TO1/7f	0,094103	0,750571	0,001046	0,005799	0,167698		0,000000	0,011729	0,000443	0,000003
	TO1/6f	0,000000	0,000000	0,000664	0,000118	0,000003	0,000000		0,000000	0,001564	0,170903
	LO5/1f	0,321941	0,005702	0,000002	0,000010	0,000436	0,011729	0,000000		0,000001	0,000000
	BF9	0,000008	0,000942	0,717274	0,280458	0,011925	0,000443	0,001564	0,000001		0,036807
	T4	0,000000	0,000006	0,016956	0,003213	0,000068	0,000003	0,170903	0,000000	0,036807	

Table S6. The percentage of biofilm formation by *C. albicans* in the presence of filtered culture of host bacteria. The results represent the averages of triplicate experiments.

**Filtered culture of host bacteria**	**Biofilm formation [%]**
Control	100
BF9/*E. coli*	99.2 ± 0.2
BF15/*E. coli*	98.3 ± 0.1
BF17/*E. coli*	98.1 ± 0.3
FD/*E. coli*	98.7 ± 0.4
Felix/S*almonella*	99.8 ± 0.1
JG004/*P. aeruginosa*	99.4 ± 0.5
LO5/1f/ *Enterobacter cloacae*	99.9 ± 0.2
T4/*E. coli*	99.5 ± 0.1
TO1/6f/ *Ent. faecalis*	98.9 ± 0.4
TO1/7f/*Ent. faecalis*	98.6 ± 0.3

Table S7. The percentage of biofilm formation by *C. albicans* **in the presence** of RLs, bacteriophages, and their combinations. The results represent the averages of triplicate experiments ± SD. The same uppercase letters designate homogenous groups comparing bacteriophages within the same treatment, and the same lowercase letters designate homogenous groups comparing control and samples within the same bacteriophages at a P-value of <0.05.

Phage	BF17	Felix	JG004	BF15	FD	TO1/7f	TO1/6f	LO5/1f	BF9	T4
Control	**100.0** $^{A}_{a}$									
RLs 50 mg/L	**23.7** ± 3.3 $^{A}_{b}$									
Bacteriophage + RLs 50 mg/L	**19.5** ± 3.0 $^{AB}_{c}$	**20.7** ± 2.1 $^{AB}_{c}$	**19.7** ± 1.5 $^{B}_{c}$	**22.2** ± 2.5 $^{D}_{b}$	**21.5** ± 0.9 $^{D}_{c}$	**18.5** ± 4.8 $^{AC}_{c}$	**17.1** ± 4.6$^{E}_{c}$	**17.8**± 4.1 $^{CE}_{c}$	**16.8** ± 5.1 $^{E}_{c}$	**15.0** ± 5.9 $^{F}_{c}$
RLs 100 mg/L	**15.0** ± 3.7 $^{A}_{d}$	**15.0** ± 3.7 $^{A}_{d}$	**15.0** ± 3.7 $^{A}_{d}$	**15.0** ± 3.7 $^{A}_{c}$	**15.0** ± 3.7 $^{A}_{d}$	**15.0** ± 3.7 $^{A}_{d}$	**15.0** ± 3.7 $^{A}_{d}$	**15.0** ± 3.7 $^{A}_{d}$	**15.0** ± 3.7 $^{A}_{c}$	**15.0** ± 3.7 $^{A}_{c}$
Bacteriophage + RLs 100 mg/L	**13.3** ± 6.1 $^{A}_{d}$	**14.3** ± 0.7 $^{F}_{d}$	**11.3**± 2.0 $^{DE}_{e}$	**14.5** ± 1.4 $^{F}_{c}$	**12.6** ± 3.3 $^{AB}_{e}$	**11.3** ± 0.6 $^{DE}_{e}$	**12.2** ± 5.1 $^{BC}_{e}$	**12.2** ± 2.9 $^{BC}_{e}$	**11.8** ± 4.1 $^{CD}_{d}$	**10.6** ± 3.2 $^{E}_{d}$
RLs 200 mg/L	**9.9** ± 4.9 $^{A}_{e}$	**9.9** ± 4.9 $^{A}_{e}$	**9.9** ± 4.9 $^{A}_{e}$	**9.9** ± 4.9 $^{A}_{d}$	**9.9** ± 4.9 $^{A}_{f}$	**9.9** ± 4.9 $^{A}_{e}$	**9.9** ± 4.9 $^{A}_{f}$	**9.9** ± 4.9 $^{A}_{f}$	**9.9** ± 4.9 $^{A}_{d}$	**9.9** ± 4.9 $^{A}_{d}$
Bacteriophage + RLs 200 mg/L	**8.5** ± 6.7 $^{A}_{e}$	**8.1** ± 2.8 $^{A}_{e}$	**5.3** ± 6.7 $^{B}_{f}$	**5.5** ± 8.5 $^{B}_{e}$	**7.1** ± 7.3 $^{DE}_{g}$	**6.6** ± 2.5 $^{CD}_{f}$	**6.2** ± 2.2 $^{C}_{g}$	**7.4** ± 4.5 $^{E}_{g}$	**5.2** ± 6.0 $^{B}_{e}$	**5.4** ± 1.4 $^{B}_{e}$
Bacteriophage (5x10^7 pfu/mL)	**93.7** ± 1.7 $^{AD}_{f}$	**90.8** ± 1.8 $^{AB}_{f}$	**86.0** ± 4.1 $^{C}_{g}$	**87.0** ± 0.6 $^{CE}_{f}$	**89.3** ± 1.3 $^{BE}_{h}$	**91.3** ± 1.0 $^{AB}_{g}$	**80.4** ± 1.0 $^{F}_{h}$	**95.1** ± 1.5 $^{D}_{h}$	**85.4** ± 2.1 $^{C}_{f}$	**82.3** ± 2.0 $^{F}_{f}$

Table S8. One-way ANOVA and least significance difference (LSD) post-hoc comparisons of means of percentage of biofilm formation by *C. albicans* **in the presence** of RLs, bacteriophages, and their combinations. Data are presented as P-values between samples for each phage. Values depicted in red are statistically significant at a 95% confidence interval.

Phage	Sample	Control	RLs 50 mg/L	RLs 100 mg/L	RLs 200 mg/L	BF + RLs 50 mg/L	BF + RLs 100 mg/L	BF + RLs 200 mg/L	Phage
BF17	Control		0,000000	0,000000	0,000000	0,000000	0,000000	0,000000	0,000008
	RLs 50 mg/L	0,000000		0,598447	0,003769	0,001771	0,000434	0,000001	0,000000
	RLs 100 mg/L	0,000000	0,598447		0,011611	0,005503	0,001335	0,000001	0,000000
	RLs 200 mg/L	0,000000	0,003769	0,011611		0,725278	0,318843	0,000264	0,000000
	BF + RLs 50 mg/L	0,000000	0,001771	0,005503	0,725278		0,511654	0,000553	0,000000
	BF + RLs 100 mg/L	0,000000	0,000434	0,001335	0,318843	0,511654		0,002269	0,000000
	BF + RLs 200 mg/L	0,000000	0,000001	0,000001	0,000264	0,000553	0,002269		0,000000
	Phage	0,000008	0,000000	0,000000	0,000000	0,000000	0,000000	0,000000	
Felix	control		0,000000	0,000000	0,000000	0,000000	0,000000	0,000000	0,007220
	RLs 50 mg/L	0,000000		0,564915	0,001927	0,001039	0,001168	0,000021	0,000000
	RLs 100 mg/L	0,000000	0,564915		0,006652	0,003591	0,004037	0,000065	0,000000
	RLs 200 mg/L	0,000000	0,001927	0,006652		0,773321	0,815315	0,039927	0,000000
	BF + RLs 50 mg/L	0,000000	0,001039	0,003591	0,773321		0,956429	0,069781	0,000000
	BF + RLs 100 mg/L	0,000000	0,001168	0,004037	0,815315	0,956429		0,062909	0,000000
	BF + RLs 200 mg/L	0,000000	0,000021	0,000065	0,039927	0,069781	0,062909		0,000000
	BF	0,007220	0,000000	0,000000	0,000000	0,000000	0,000000	0,000000	
JG004	control		0,000000	0,000000	0,000000	0,000000	0,000000	0,000000	0,000000
	RLs 50 mg/L	0,000000		0,334651	0,000011	0,000002	0,000000	0,000000	0,000000
	RLs 100 mg/L	0,000000	0,334651		0,000076	0,000014	0,000000	0,000000	0,000000
	RLs 200 mg/L	0,000000	0,000011	0,000076		0,404535	0,000025	0,000000	0,000000

	BF + RLs 50 mg/L	0,000000	0,000002	0,000014	0,404535		0,000136	0,000000	0,000000
	BF + RLs 100 mg/L	0,000000	0,000000	0,000000	0,000025	0,000136		0,002306	0,000000
	BF + RLs 200 mg/L	0,000000	0,000000	0,000000	0,000000	0,000000	0,002306		0,000000
	BF	0,000000	0,000000	0,000000	0,000000	0,000000	0,000000	0,000000	
**BF15**	control		0,000000	0,000000	0,000000	0,000000	0,000000	0,000000	0,226943
	RLs 50 mg/L	0,000000		0,751245	0,059057	0,040100	0,026264	0,003320	0,000000
	RLs 100 mg/L	0,000000	0,751245		0,106606	0,074011	0,049443	0,006546	0,000000
	RLs 200 mg/L	0,000000	0,059057	0,106606		0,842700	0,683034	0,176572	0,000000
	BF + RLs 50 mg/L	0,000000	0,040100	0,074011	0,842700		0,833118	0,243053	0,000000
	BF + RLs 100 mg/L	0,000000	0,026264	0,049443	0,683034	0,833118		0,333147	0,000000
	BF + RLs 200 mg/L	0,000000	0,003320	0,006546	0,176572	0,243053	0,333147		0,000000
	BF	0,226943	0,000000	0,000000	0,000000	0,000000	0,000000	0,000000	
**FD**	control		0,000000	0,000000	0,000000	0,000000	0,000000	0,000000	0,000000
	RLs 50 mg/L	0,000000		0,310323	0,000006	0,000000	0,000000	0,000000	0,000000
	RLs 100 mg/L	0,000000	0,310323		0,000044	0,000000	0,000000	0,000000	0,000000
	RLs 200 mg/L	0,000000	0,000006	0,000044		0,000246	0,000003	0,000000	0,000000
	BF + RLs 50 mg/L	0,000000	0,000000	0,000000	0,000246		0,030522	0,000091	0,000000
	BF + RLs 100 mg/L	0,000000	0,000000	0,000000	0,000003	0,030522		0,012559	0,000000
	BF + RLs 200 mg/L	0,000000	0,000000	0,000000	0,000000	0,000091	0,012559		0,000000
	BF	0,000000	0,000000	0,000000	0,000000	0,000000	0,000000	0,000000	
**TO1/7f**	control		0,000000	0,000000	0,000000	0,000000	0,000000	0,000000	0,000000
	RLs 50 mg/L	0,000000		0,537787	0,001103	0,000275	0,000080	0,000001	0,000000
	RLs 100 mg/L	0,000000	0,537787		0,004167	0,001021	0,000287	0,000002	0,000000
	RLs 200 mg/L	0,000000	0,001103	0,004167		0,514445	0,220186	0,000952	0,000000
	BF + RLs 50 mg/L	0,000000	0,000275	0,001021	0,514445		0,550933	0,003886	0,000000
	BF + RLs 100 mg/L	0,000000	0,000080	0,000287	0,220186	0,550933		0,013874	0,000000
	BF + RLs 200 mg/L	0,000000	0,000001	0,000002	0,000952	0,003886	0,013874		0,000000

	BF	0,000007	0,000000	0,000000	0,000000	0,000000	0,000000	0,000000	
**TO1/6f**	control		0,000000	0,000000	0,000000	0,000000	0,000000	0,000000	0,000000
	RLs 50 mg/L	0,000000		0,437649	0,000127	0,000033	0,000002	0,000000	0,000000
	RLs 100 mg/L	0,000000	0,437649		0,000650	0,000158	0,000010	0,000000	0,000000
	RLs 200 mg/L	0,000000	0,000127	0,000650		0,502296	0,052080	0,000035	0,000000
	BF + RLs 50 mg/L	0,000000	0,000033	0,000158	0,502296		0,177043	0,000137	0,000000
	BF + RLs 100 mg/L	0,000000	0,000002	0,000010	0,052080	0,177043		0,002583	0,000000
	BF + RLs 200 mg/L	0,000000	0,000000	0,000000	0,000035	0,000137	0,002583		0,000000
	BF	0,000000	0,000000	0,000000	0,000000	0,000000	0,000000	0,000000	
**LO5/1f**	control		0,000000	0,000000	0,000000	0,000000	0,000000	0,000000	0,000000
	RLs 50 mg/L	0,000000		0,449062	0,000163	0,000008	0,000140	0,000000	0,000000
	RLs 100 mg/L	0,000000	0,449062		0,000812	0,000032	0,000691	0,000000	0,000000
	RLs 200 mg/L	0,000000	0,000163	0,000812		0,130752	0,939672	0,000000	0,000000
	BF + RLs 50 mg/L	0,000000	0,000008	0,000032	0,130752		0,149030	0,000001	0,000000
	BF + RLs 100 mg/L	0,000000	0,000140	0,000691	0,939672	0,149030		0,000000	0,000000
	BF + RLs 200 mg/L	0,000000	0,000000	0,000000	0,000000	0,000001	0,000000		0,000000
	BF	0,000000	0,000000	0,000000	0,000000	0,000000	0,000000	0,000000	
**BF9**	control		0,000000	0,000000	0,000000	0,000000	0,000000	0,000000	0,000007
	RLs 50 mg/L	0,000000		0,438932	0,000130	0,001587	0,000100	0,000000	0,000000
	RLs 100 mg/L	0,000000	0,438932		0,000666	0,008446	0,000504	0,000001	0,000000
	RLs 200 mg/L	0,000000	0,000130	0,000666		0,245102	0,895123	0,003875	0,000000
	BF + RLs 50 mg/L	0,000000	0,001587	0,008446	0,245102		0,198777	0,000308	0,000000
	BF + RLs 100 mg/L	0,000000	0,000100	0,000504	0,895123	0,198777		0,005138	0,000000
	BF + RLs 200 mg/L	0,000000	0,000000	0,000001	0,003875	0,000308	0,005138		0,000000
	BF	0,000007	0,000000	0,000000	0,000000	0,000000	0,000000	0,000000	
**T4**	control		0,000000	0,000000	0,000000	0,000000	0,000000	0,000000	0,000000

RLs 50 mg/L	0,000000		0,428629	0,000103	0,000604	0,000025	0,000000	0,000000
RLs 100 mg/L	0,000000	0,428629		0,000544	0,003340	0,000124	0,000000	0,000000
RLs 200 mg/L	0,000000	0,000103	0,000544		0,401194	0,482445	0,001697	0,000000
BF + RLs 50 mg/L	0,000000	0,000604	0,003340	0,401194		0,133319	0,000280	0,000000
BF + RLs 100 mg/L	0,000000	0,000025	0,000124	0,482445	0,133319		0,007721	0,000000
BF + RLs 200 mg/L	0,000000	0,000000	0,000000	0,001697	0,000280	0,007721		0,000000
BF	0,000000	0,000000	0,000000	0,000000	0,000000	0,000000	0,000000	

Table S9. One-way ANOVA and least significance difference (LSD) post-hoc comparisons of means of percentage of biofilm formation by *C. albicans* **in the presence** of RLs, bacteriophages, and their combinations. Data are presented as P-values between phages for each sample. Values depicted in red are statistically significant at a 95% confidence interval.

Sample	Phage	BF17	Felix	JG004	BF15	FD	TO1/7f	TO1/6f	LO5/1f	BF9	T4
**BF + RLs 50 mg/L**	BF17		0,937641	0,927276	0,984972	0,086103	0,854065	0,926701	0,518495	0,312657	0,422626
	Felix	0,937641		0,865433	0,952631	0,074109	0,793302	0,864864	0,469975	0,350203	0,468232
	JG004	0,927276	0,865433		0,912329	0,102194	0,926125	0,999422	0,578458	0,272575	0,373097
	BF15	0,984972	0,952631	0,912329		0,083070	0,839336	0,911755	0,506565	0,321425	0,433342
	FD	0,086103	0,074109	0,102194	0,083070		0,121112	0,102332	0,264500	0,010094	0,016260
	TO1/7f	0,854065	0,793302	0,926125	0,839336	0,121112		0,926701	0,642771	0,235868	0,326906
	TO!/6F	0,926701	0,864864	0,999422	0,911755	0,102332	0,926701		0,578947	0,272273	0,372720
	LO5/1f	0,518495	0,469975	0,578458	0,506565	0,264500	0,642771	0,578947		0,105960	0,155531
	BF9	0,312657	0,350203	0,272575	0,321425	0,010094	0,235868	0,272273	0,105960		0,830302
	T4	0,422626	0,468232	0,373097	0,433342	0,016260	0,326906	0,372720	0,155531	0,830302	
**BF + RLs 100 mg/L**	BF17		0,253897	0,005987	0,632065	0,001256	0,931803	0,581104	0,173364	0,189685	0,432879
	Felix	0,253897		0,000393	0,499064	0,000081	0,221680	0,098013	0,814980	0,856678	0,712100
	JG004	0,005987	0,000393		0,001961	0,505764	0,007279	0,020644	0,000226	0,000257	0,000943
	BF15	0,632065	0,499064	0,001961		0,000403	0,573081	0,307520	0,365695	0,393863	0,756664
	FD	0,001256	0,000081	0,505764	0,000403		0,001537	0,004592	0,000047	0,000053	0,000194
	TO1/7f	0,931803	0,221680	0,007279	0,573081	0,001537		0,640474	0,149619	0,164125	0,385584
	TO!/6F	0,581104	0,098013	0,020644	0,307520	0,004592	0,640474		0,062509	0,069425	0,188560
	LO5/1f	0,173364	0,814980	0,000226	0,365695	0,000047	0,149619	0,062509		0,957343	0,547795
	BF9	0,189685	0,856678	0,000257	0,393863	0,000053	0,164125	0,069425	0,957343		0,583521
	T4	0,432879	0,712100	0,000943	0,756664	0,000194	0,385584	0,188560	0,547795	0,583521	
	BF17		0,000015	0,336954	0,000069	0,386059	0,016064	0,085299	0,001095	0,000048	0,000135
	Felix	0,000015		0,000002	0,506547	0,000002	0,006360	0,000961	0,000000	0,609429	0,343944

**BF + RLs 200 mg/L**	JG004	0,336954	0,000002		0,000007	0,923175	0,001734	0,011201	0,010423	0,000005	0,000014
	BF15	0,000069	0,506547	0,000007		0,000009	0,027901	0,004598	0,000000	0,876555	0,772558
	FD	0,386059	0,000002	0,923175	0,000009		0,002174	0,013885	0,008385	0,000007	0,000018
	TO1/7f	0,016064	0,006360	0,001734	0,027901	0,002174		0,422298	0,000003	0,019987	0,050803
	TO!/6F	0,085299	0,000961	0,011201	0,004598	0,013885	0,422298		0,000017	0,003206	0,008909
	LO5/1f	0,001095	0,000000	0,010423	0,000000	0,008385	0,000003	0,000017		0,000000	0,000000
	BF9	0,000048	0,609429	0,000005	0,876555	0,000007	0,019987	0,003206	0,000000		0,657325
	T4	0,000135	0,343944	0,000014	0,772558	0,000018	0,050803	0,008909	0,000000	0,657325	
**BF**	BF17		0,082647	0,670534	0,040484	0,028312	0,566338	0,052387	0,006546	0,316654	0,150424
	Felix	0,082647		0,035216	0,719932	0,000450	0,025690	0,000911	0,000095	0,433089	0,003396
	JG004	0,670534	0,035216		0,016312	0,067624	0,881218	0,118587	0,017025	0,160142	0,300168
	BF15	0,040484	0,719932	0,016312		0,000193	0,011714	0,000389	0,000041	0,258244	0,001464
	FD	0,028312	0,000450	0,067624	0,000193		0,090132	0,766196	0,510175	0,002901	0,395388
	TO1/7f	0,566338	0,025690	0,881218	0,011714	0,090132		0,154607	0,023547	0,123032	0,372483
	TO1/6f	0,052387	0,000911	0,118587	0,000389	0,766196	0,154607		0,342667	0,005780	0,576935
	LO5/1f	0,006546	0,000095	0,017025	0,000041	0,510175	0,023547	0,342667		0,000609	0,139448
	BF9	0,316654	0,433089	0,160142	0,258244	0,002901	0,123032	0,005780	0,000609		0,020238
	T4	0,150424	0,003396	0,300168	0,001464	0,395388	0,372483	0,576935	0,139448	0,020238	

Table S10. One-way ANOVA and least significance difference (LSD) post-hoc comparisons of means of expression of genes responsible for biofilm formation in *Candida* yeast treated with RLs, phages and their combinations. Data are presented as P-values between samples for each phage. Values depicted in red are statistically significant at a 95% confidence interval.

		**HWP1**			
**BF17**		Control	Phage	RLs 200 mg/L	RLs+BF
	Control		0,020776	0,000050	0,000039
	Phage	0,020776		0,000121	0,000088
	RLs 200mg/L	0,000050	0,000121		0,284606
	RLs+BF	0,000039	0,000088	0,284606	
**LO5/1f**		Control	Phage	RLs 200 mg/L	RLs+BF
	Control		0,000870	0,000006	0,000002
	Phage	0,000870		0,000023	0,000004
	RLs 200mg/L	0,000006	0,000023		0,000248
	RLs+BF	0,000002	0,000004	0,000248	
**JG004**		Control	Phage	RLs 200 mg/L	RLs+BF
	Control		0,015585	0,000065	0,000028
	Phage	0,015585		0,000186	0,000065
	RLs 200mg/L	0,000065	0,000186		0,015585
	RLs+BF	0,000028	0,000065	0,015585	
**FD**		Control	Phage	RLs 200 mg/L	RLs+BF
	Control		0,000574	0,000008	0,000004
	Phage	0,000574		0,000044	0,000017
	RLs 200mg/L	0,000008	0,000044		0,007016
	RLs+BF	0,000004	0,000017	0,007016	
		**ALS3**			
**BF17**		Control	Phage	RLs 200 mg/L	RLs+BF
	Control		0,004524	0,000059	0,000027

	Phage	0,004524		0,000276	0,000094
	RLs 200mg/L	0,000059	0,000276		0,019416
	RLs+BF	0,000027	0,000094	0,019416	

**LO5/1f**		Control	Phage	RLs 200 mg/L	RLs+BF
	Control		0,000384	0,000009	0,000002
	Phage	0,000384		0,000065	0,000006
	RLs 200mg/L	0,000009	0,000065		0,000155
	RLs+BF	0,000002	0,000006	0,000155	

**JG004**		Control	Phage	RLs 200 mg/L	RLs+BF
	Control		0,002731	0,000071	0,000023
	Phage	0,002731		0,000488	0,000092
	RLs 200mg/L	0,000071	0,000488		0,005329
	RLs+BF	0,000023	0,000092	0,005329	

**FD**		Control	Phage	RLs 200 mg/L	RLs+BF
	Control		0,005706	0,000047	0,000026
	Phage	0,005706		0,000179	0,000082
	RLs 200mg/L	0,000047	0,000179		0,041582
	RLs+BF	0,000026	0,000082	0,041582	

ECE1

**BF17**		Control	Phage	RLs 200 mg/L	RLs+BF
	Control		0,008537	0,000007	0,000005
	Phage	0,008537		0,000013	0,000010
	RLs 200mg/L	0,000007	0,000013		0,183567
	RLs+BF	0,000005	0,000010	0,183567	

**LO5/1f**		Control	Phage	RLs 200 mg/L	RLs+BF

	Control		0,001221	0,000008	0,000003
	Phage	0,001221		0,000028	0,000008
	RLs 200mg/L	0,000008	0,000028		0,001103
	RLs+BF	0,000003	0,000008	0,001103	

**JG004**		Control	Phage	RLs 200 mg/L	RLs+BF
	Control		0,002949	0,000011	0,000007
	Phage	0,002949		0,000033	0,000019
	RLs 200mg/L	0,000011	0,000033		0,038763
	RLs+BF	0,000007	0,000019	0,038763	

**FD**		Control	Phage	RLs 200 mg/L	RLs+BF
	Control		0,003789	0,000004	0,000003
	Phage	0,003789		0,000008	0,000005
	RLs 200mg/L	0,000004	0,000008		0,021268
	RLs+BF	0,000003	0,000005	0,021268	

SAP4

**BF17**		Control	Phage	RLs 200 mg/L	RLs+BF
	Control		0,013236	0,000018	0,000012
	Phage	0,013236		0,000038	0,000025
	RLs 200mg/L	0,000018	0,000038		0,077916
	RLs+BF	0,000012	0,000025	0,077916	

**LO5/1f**		Control	Phage	RLs 200 mg/L	RLs+BF
	Control		0,001056	0,000016	0,000003
	Phage	0,001056		0,000084	0,000007
	RLs 200mg/L	0,000016	0,000084		0,000139
	RLs+BF	0,000003	0,000007	0,000139	

JG004	Control	Phage	RLs 200 mg/L	RLs+BF
Control		0,005692	0,000034	0,000013
Phage	0,005692		0,000115	0,000032
RLs 200mg/L	0,000034	0,000115		0,004395
RLs+BF	0,000013	0,000032	0,004395	

FD	Control	Phage	RLs 200 mg/L	RLs+BF
Control		0,025889	0,000198	0,000095
Phage	0,025889		0,000657	0,000252
RLs 200mg/L	0,000198	0,000657		0,044728
RLs+BF	0,000095	0,000252	0,044728	

Table S11. Chemical structure and relative abundance of RLs used in this work.

RL homologue	Relative abundance [%]
Rha-C_{10}-C_{10}	39.5
Rha-Rha-C_{10}-C_{10}	15.9
Rha-C_{10}	14.2
Rha-C_{10}-C_8/<br>Rha-C_8-C_{10}	9.5
Rha-Rha-C_{10}	7.8
Rha-C_{10}-$C_{12:1}$/<br>Rha-$C_{12:1}$-C_{10}	6.5
Rha-C_{10}-C_{12}/<br>Rha-C_{12}-C_{10}	6.6

Table S12. Characteristics of bacteriophages used in the studies.

Bacteriophage	Morphotype	Bacterial host	Genome accession number	Life cycle	Exemplary literature
**T4**[a] (DSM 4505)	*Myoviridae*	*Escherichia coli* (DSM-613)	NC_000866	virulent, lytic cycle	[49,50]
**JG004**[a] (DSM 19871)	*Myoviridae*	*Pseudomonas aeruginosa* (DSM-19880)	NC_019450	virulent, lytic cycle	[51]
**Felix 01**[a] (DSM 18524)	*Myoviridae*	*Salmonella enterica* (DSM-18522)	AF320576 NC_005282	virulent, lytic cycle	[52]
**FD**[a] (DSM 4498)	*Inoviridae*	*Escherichia coli* (DSM 8226)	NC_025824 .1	Temperate, non-lytic	[53]
**BF9**[b]	*Siphoviridae*	*Escherichia coli*	MW822006	virulent, lytic cycle	[54]
**BF15**[b]	*Myoviridae*	*Escherichia coli*	MW822007	virulent, lytic cycle	[54]
**BF17**[b]	*Siphoviridae*	*Escherichia coli*	MW822008	virulent, lytic cycle	[54]
**LO5+1F**[b]	*Myoviridae*	*Enterobacter cloacae*	unavailable	virulent, lytic cycle	Data regarding phage characteristics unpublished
**TO1+6F**[b]	*Siphoviridae*	*Enterococcus faecalis*	unavailable	virulent, lytic cycle	Data regarding phage

					characteristics unpublished
**TO1+7F**[b]	*Siphoviridae*	*Enterococcus faecalis*	unavailable	virulent, lytic cycle	Data regarding phage characteristics unpublished

[a] Leibniz Institute DSMZ-German Collection of Microorganisms and Cell Cultures

[b] Collection of Microorganisms of Department of Biotechnology and Environmental Microbiology, Wrocław University of Environmental and Life Sciences

Table S13. List of primers used for qRT-PCR experiments [47].

**Primer**	**Sequence (5'-3')**
ACT1-F	GGTTTGGAAGCTGCTGGTATTGACC
ACT1-R	ACGTTCAGCAATACCTGGGAACATG
ALS3-F	CTAATGCTGCTACGTATAATT
ALS3-R	CCTGAAATTGACATGTAGCA
ECE1-F	GCTGGTATCATTGCTGATAT
ECE1-R	TTCGATGGATTGTTGAACAC
HWP1-F	TGGTGCTATTACTATTCCGG
HWP1-R	CAATAATAGCAGCACCGAAG
SAP4-F	GGTACCGTTGATTTCCAATTC
SAP4-R	ATCTTCACTTTCACGAACACG
